# The Impact of WHO Essential Medicines Policies on Inappropriate Use of Antibiotics

**DOI:** 10.1371/journal.pone.0152020

**Published:** 2016-03-22

**Authors:** Kathleen Anne Holloway, Laura Rosella, David Henry

**Affiliations:** 1 Department of Health Systems Development, World Health Organization, Regional Office SouthEast Asia, New Delhi, India; 2 Epidemiology Division, Dalla Lana School of Public Health, The University of Toronto, Toronto, Canada; University of Hyderabad, INDIA

## Abstract

**Background:**

Inappropriate overuse of antibiotics contributes to antimicrobial resistance (AMR), yet policy implementation to reduce inappropriate antibiotic use is poor in low and middle-income countries.

**Aims:**

To determine whether public sector inappropriate antibiotic use is lower in countries reporting implementation of selected essential medicines policies.

**Materials and Methods:**

Results from independently conducted antibiotic use surveys in countries that did, and did not report implementation of policies to reduce inappropriate antibiotic prescribing, were compared. Survey data on four validated indicators of inappropriate antibiotic use and 16 self-reported policy implementation variables from WHO databases were extracted. The average difference for indicators between countries reporting versus not reporting implementation of specific policies was calculated. For 16 selected policies we regressed the four antibiotic use variables on the numbers of policies the countries reported implementing.

**Results:**

Data were available for 55 countries. Of 16 policies studied, four (having a national Ministry of Health unit on promoting rational use of medicines, a national drug information centre and provincial and hospital drugs and therapeutics committees) were associated with statistically significant reductions in antibiotic use of ≥20% in upper respiratory infection (URTI). A national strategy to contain antibiotic resistance was associated with a 30% reduction in use of antibiotics in acute diarrheal illness. Policies seemed to be associated with greater effects in antibiotic use for URTI and diarrhea compared with antibiotic use in all patients. There were negative correlations between the numbers of policies reported implemented and the percentage of acute diarrhoea cases treated with antibiotics (r = -0.484, p = 0.007) and the percentage of URTI cases treated with antibiotics (r = -0.472, p = 0.005). Major study limitations were the reliance on self-reported policy implementation data and antibiotic use data from linited surveys.

**Conclusions:**

Selected essential medicines policies were associated with lower antibiotic use in low and middle income countries.

## Introduction

Antibiotic drug resistance is a serious global public health problem with high human and financial costs.[1–6] Inappropriate and over use of antibiotic drugs is widespread and contributes to rapidly increasing bacterial resistance.[7,8]

Policy-relevant interventions to combat inappropriate medicines use have generally had small effects.[9] In the case of antibiotic drugs this may be because the determinants of use, particularly in developing countries, have not been sufficiently understood or addressed.[10] The World Health Organisation (WHO) has long advocated for introduction of essential medicines policies to encourage rational use of medicines.[11, 12] These include specific measures to promote prudent use of antibiotic drugs to minimize the development of resistance.[13–15] However, implementation of policies in many low and middle-income countries is suboptimal with less than half of countries implementing many recommended policies.[16, 17]

In Europe, coordinated medicine policy implementation resulted in reduced inappropriate antibiotic use [18–20], but there is less evidence of policy impact in developing countries. [21] There is emerging evidence from analyses of WHO databases that some policies aimed at improving use of medicines generally are associated with more rational use and that the impact increases with the numbers of policies that are implemented at country level. [22]

The aim of this study was to perform further analyses of the WHO databases to determine if inappropriate antibiotic use in the public sector is less in low and middle-income countries that report implementing essential medicines policies than in those that do not.

## Materials and Methods

Our overall methods have been described in detail previously.[22] In the previous study we evaluated a full range of policy options and a broad set of medicines use indicators. Here we focused on policies that are most relevant to prescribing of antibiotics and selected specific antibiotic use indicators. The study was limited to the public sector due to the lack of medicines use data in the private sector. We did not have access to time series data that would enable longitudinal analysis of policy impacts.

A dataset of antibiotic use (outcome) and reported policy implementation (exposure), with one set of policy implementation data and antibiotic use for each country, was created from two WHO databases. Public sector antibiotic use data for 2002–8, by country, were extracted from the WHO medicines use database.[7, 23, 24] The database contains information on medicines use in primary care in developing and transitional countries extracted from survey reports. We included only survey reports that described use of recommended validated measures of medicines use estimated from at least 600 prescriptions and/or three or more facilities.[25, 26] Policy implementation data were extracted from WHO policy databases of questionnaires sent to Ministries of Health in 2003 and 2007.[16, 17] In these surveys, questions on pharmaceutical policy and regulation are asked in a standard format [27].

### Variables

We excluded policies that had previously been shown to have no effect on overall medicines use, and overlapping policies, as described previously.[22] Of the remainder we selected policies that we considered most relevant to antibiotic use. In doing this we were guided by the WHO Global Strategy for Containment of Antimicrobial Resistance (AMR) and the WHO Global Action plan on AMR.[13, 28, 29] Those policies that best captured recommended strategies to reduce inappropriate antibiotic use and contain AMR were selected.[13, 28, 29] In this way, 16 policies were evaluated. They are listed in Table 1.

We chose four outcome variables [25, 26]:

- % of primary care cases receiving antibiotics. This gives some indication of overall antibiotic use
- % of upper respiratory tract infection cases that received antibiotics (high values signify over-use)
- % acute diarrhea cases that received antibiotics (high values signify over-use)
- % cases not needing antibiotics that received antibiotics (inappropriate use)

**Table 1:** Differences in antibiotic use between countries with and without each of 16 policies hypothesized to decrease inappropriate antibiotic use.

	National medicines policies and strategies (numbers in parenthesis refer to the number of countries contributing data to each analysis)	% of primary care cases receiving antibiotics	% of upper respiratory infection cases given ABs	% of acute diarrhea cases that received antibiotics	% of cases not needing antibiotics that received them (inappropriate use)
	**National policies**				
1	National strategy to contain antibiotic resistance (n = 35, 25, 23, 18)	-3.4	-15.3*	-30.7*	-2.7
	Educational policies				
2	Undergraduate training of doctors on the Standard Treatment Guidelines (n = 28, 21, 20, 17)	-4.1	-17.9	-13.5	-7.5
3	Undergraduate training of nurses on the Standard Treatment Guidelines (n = 27, 20, 20, 16)	-1.9	-14.3	-5.8	-7.0
4	Public education on antibiotics in last 2 years (n = 38, 28, 27, 17)	-1.1	-19.5	-8.8	-4.0
	**Managerial Policies**				
5	National Essential Medicines List updated in the last 2 years (n = 32, 24, 23, 17)	-3.9	-8.6	-3.7	-7.6
6	National Formulary updated in the last 5 years (n = 43, 31, 28, 22)	-4.8	-11.9	-4.9	-2.4
	Economic Policies				
7	No Drug sales revenue used to supplement prescriber income^ (n = 40, 29, 28, 20)	-2.6	-7.6	-13.7*	-11.9
8	Drugs dispensed free of charge to all patients (n = 40, 29, 26, 20)	-6.4	-12.5	-18.8*	-19.7
9	Drugs dispensed free of charge to patients < 5 years (n = 38, 28, 24, 18)	-4.5	-11.3	-10.0	-13.5
	**Regulatory policies**				
10	Antibiotics not available over-the-counter* (n = 41, 30, 28, 21)	-1.8	+2.2	-12.1	-11.3
11	Joint regulation of drug promotion by government and industry (as opposed to regulation by government alone) (n = 40, 29, 27, 21)	-2.0	-5.0	-3.0	-13.0
	**Administrative/Structural policies**				
12	National MOH unit on promoting rational use of medicines (n = 35, 26, 25, 21)	-5.1	-22.2*	-18.1*	+4.2
13	Half or more of all general hospitals have a Drug and Therapeutic Committee^ (n = 36, 26, 25, 18)	-0.9	-25.3*	-1.4	-7.1
14	Half or more of all provinces/districts have a Drug and Therapeutic Committee^ (n = 35, 25, 23, 19)	-8.2	-21.2	-0.5	-1.4
15	Presence of National Drug Information Centre (n = 37, 27, 24, 21)	-10.2	-25.1*	-6.9	-8.5
	**Human resource policies**				
16	No prescribing by staff with less than one month's training in public primary care^ (n = 35, 25, 24, 17)	-2.6	-3.1	-5.4	+0.1

^ Graded response converted to a “yes/no” response.

* p< 0.05. Note: these P values are not corrected for multiple testing and are presented here to help identify patterns in the data not to test hypotheses regarding the relative effectiveness of the different policies.

As national wealth has been shown to correlate with policy implementation and quality use of medicines economic status was considered a potential confounder.^22^ Gross National Income per capita (GNI per capita) in 2009/2010 for countries was extracted from the World Bank data-base http://data.worldbank.org/indicator/NY.GDP.PCAP.CD and used to stratify some analyses (see below).

### Analyses

Univariate analysis was used to estimate the differences in average scores (as proportions reporting the outcome) for each of the four antibiotic use indicators between countries that did or did not report implementation of the 16 individual policies. Each policy was the unit of analysis and for each of these indicators a lower score equated with better use. The sample sizes were too small to enable direct comparisons of different policy impacts, so the effect sizes reported here cannot be used to makes inferences about relative effectiveness of the policies we evaluated. Consequently, we did not adjust the P values for multiple testing. To look at the impact of multiple policies we regressed the scores for each of the 4 outcome measures on the number (out of 16) of policies countries reported implementing. Because of missing data we had to adjust the estimated numbers of implemented policies using the formula: adjusted policy number = (number of policies reported /(16—missing values for policies))*16.[22] In these analyses each country was the unit of analysis. We examined the impact of national wealth by performing separate regressions of outcomes on policy numbers separately for countries above and below the median value for gross national income per capita (GNIpc). All analyses were done in Stats Direct (version 2.7.9) and SAS (version 9.4).

## Results

From the WHO database, 55 countries had analysable data on policies and antibiotic use. Out of a possible 880 policy responses (16 policies in each of 55 countries), 718 (82%) were available for analysis.

### Comparison of Antibiotic Use Indicators in Countries With and Without Specific Policies

Table 1 shows the differences between countries that did or did not report implementation of specific policies for the four antibiotic indicators. The effect sizes associated with various policies were generally larger when measured by the two ‘condition-specific’ indicators (inappropriate use of antibiotics in upper respiratory tract infection and diarrhea) than when measured by the % patients receiving antibiotics or % of cases not needing antibiotics who received them.

Policies associated with statistically significant 20% or greater reductions in use of antibiotics in one or more of 4 settings described in the outcome indicators were: having a MOH department to promote rational use of medicines, having a national Drug Information Centre, and having drug and therapeutic committees in more than half of all general hospitals and more than half of all provinces. Several of the other policies were associated with lower use of antibiotics across all indicators, though the effects were smaller. Only 2 of 64 comparisons were associated with increased antibiotic use and the effects were small (2 and 4% respectively).

### Effects of Multiple Policies and Impact of National Wealth

**Fig 1:**
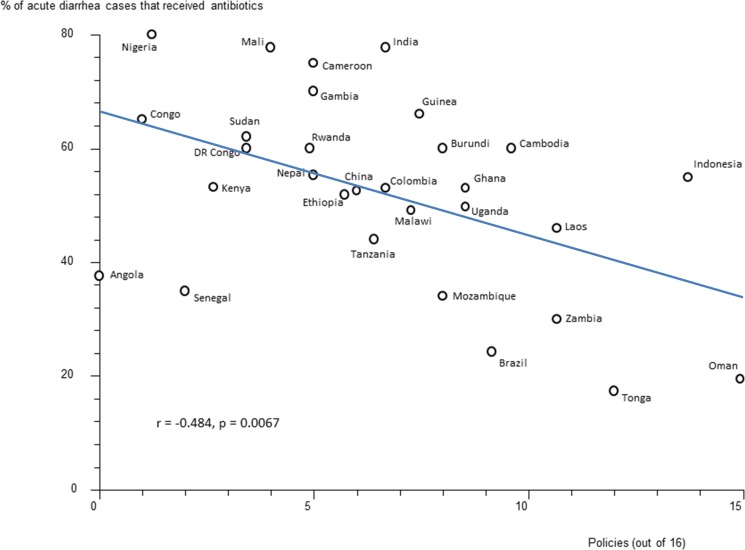
Correlation between the number of implemented policies (out of 16) and the treatment of acute diarrhea with antibiotics. Correlation coefficient (r) = -0.484, p = 0.0067. Each data point (circle) represents a country.

**Fig 2:**
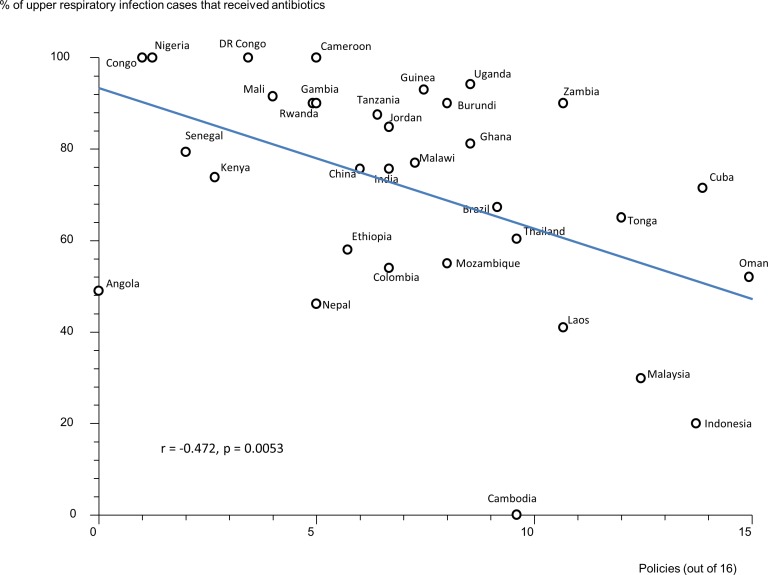
Correlation between the number of implemented policies (out of 16) and the treatment of acute upper respiratory tract infection with antibiotics. Correlation coefficient (r) = -0.472, p = 0.0053. Each data point (circle) represents a country.

**Fig 3:**
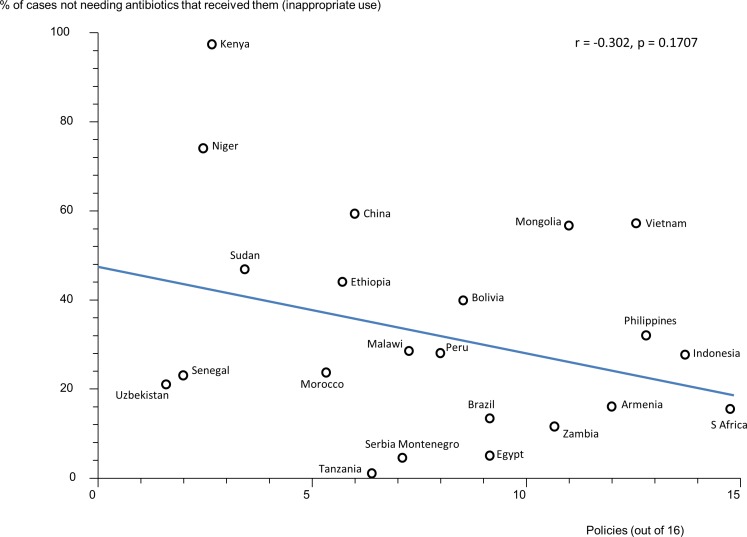
Correlation between the number of implemented policies (out of 16) and the percentage of patients not needing antibiotics who received them. Correlation coefficient (r) = -0.302, p = 0.1707. Each data point (circle) represents a country.

**Fig 4:**
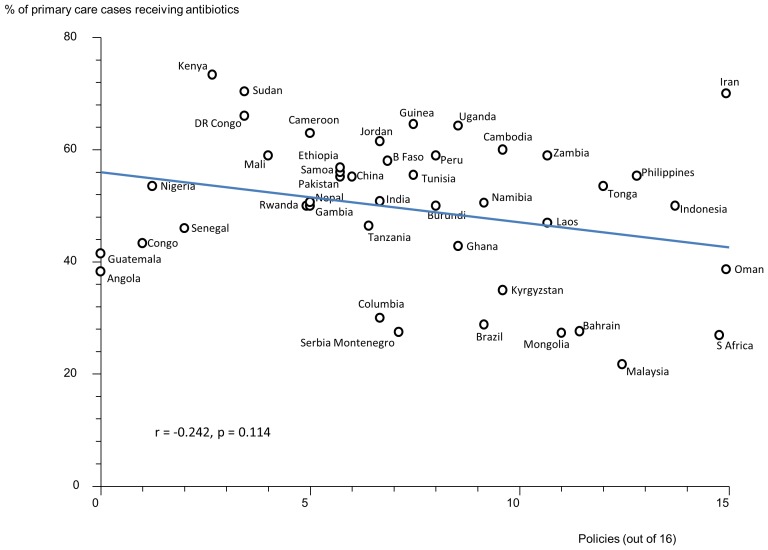
Correlation between the number of implemented policies (out of 16) and the percentage of all patients treated with antibiotics. Correlation coefficient (r) = -0.242, p = 0.114. Each data point (circle) represents a country.

There were statistically significant, moderate, negative correlations between the reported number of medicines policies implemented (out of 16) and the percentage of acute diarrhoea cases treated with antibiotics (Fig 1) and the percentage of upper respiratory tract infection cases treated with antibiotics (Fig 2). There were statistically non-significant correlations between the reported number of medicines policies implemented and the percentage of patients not needing antibiotics who received them (Fig 3) and the percentage of all patients treated with antibiotics (Fig 4).

We examined the effects of national wealth measures on these correlations. In the case of diarrheal disease, the negative correlation between numbers of policies and antibiotic use was stronger in countries below the median GNIpc ($1365) (r = -0.5860, P 0.022) than in countries above the median (r = -4821, P 0.067). However, this pattern was not seen with upper respiratory infection, where the negative correlation between policy numbers and antibiotic use was stronger in countries with GNIpc values above the median (r = -0.6008, P 0.014) than in countries with wealth levels below the median (r = -0.3407, P = 0.181).

## Discussion

A number of policies aimed at improving antibiotic use, as recommended by the WHO Global Strategy to Contain Antibiotic Resistance (AMR) and the Global Action Plan on AMR, were associated with reduced inappropriate use of antibiotics.[13, 28, 29] Policies with the most clear-cut effects were: having a MOH department dedicated to promoting rational use of medicines, having a national strategy to contain antimicrobial resistance, having a national drug information centre and having drug and therapeutic committees in hospitals and provinces. Of 64 possible comparisons of single policy effects 24 were associated with reductions in antibiotic use of 10% or more.

Effect sizes for the two condition-specific indicators of antibiotic prescribing quality (in diarrheal disease and upper respiratory infection) were greater than the effect size for the “general” indicator of antibiotic usage. These were negative correlations and the weaker association for the general indicator may be because lower use is not always better in all patients, in contrast to the specific situations of upper respiratory infection and diarrheal disease.

Countries that reported implementing more of the selected policies had lower antibiotic use, particularly in the case of upper respiratory infection and in diarrheal disease. We found that this negative correlation was stronger in low income countries in the case of diarrheal disease but this trend was not seen for URTI. This may indicate genuine differences in the two different disease settings. Diarrheal disease has been a prominent target of public health campaigns in low income countries, including promotion of oral rehydration solutions, rather than antibiotics. Alternatively, the apparent differences may be due to the play of chance because of relatively small sample sizes. Previously, when we analysed the impact of national wealth on the impacts of multiple policies on composite measures of medicines use in a larger number of countries the effects were consistently greater in low income countries.[22]

Higher socio-economic status has been previously reported to be associated with better prescribing quality [7–9, 22–24] and increased policy implementation. [16, 17, 22] Increased economic status may improve and lower antibiotic use through having healthier populations and better health systems or by making it easier to implement policies to promote better use.

The majority of interventions, described in the literature, to improve antibiotic use in primary care are educational in nature, and many have been shown to be effective in producing short-term improvements in antibiotic use.[9, 23, 24] In this study, undergraduate education of doctors and nurses on standard treatment guidelines and public education on antibiotic use were associated with lower antibiotic use in diarrheal disease and upper respiratory infection. Likewise, public education campaigns in Europe have also been effective.[18]

Administrative policies, such as a dedicated MOH department to promote rational use of medicines, having drugs and therapeutic committees in hospitals and a national drug information centre, were also associated with significantly better quality of antibiotic prescribing in our study as was the provision of free essential medicines. Administrative and managerial strategies to support decision-making have been found effective elsewhere in improving antibiotic use.[30–32] A national MOH unit dedicated to promoting rational use of medicines was associated with improved use in Oman[33], and drug and therapeutic committees with improved guideline compliance in Laos.[34]

Other policies associated with better and lower antibiotic prescribing included no over-the-counter antibiotic availability, no prescriber income from antibiotic sales, no unqualified prescribers, and joint regulation by government and industry of drug promotion. Enforcement of non-availability of antibiotics over-the-counter was associated with decreased antibiotic use in Chile [35] and reduction of prescriber income from medicine sales, through separation of prescriber-dispenser function, was associated with reduced inappropriate antibiotic use in Korea. [36] Prescriber income from medicine sales associated with worse medicines use has been seen in Zimbabwe and China respectively.[37, 38] Joint government and industry regulation has been found to be associated with better overall medicines use compared to government regulation alone.[22]

Interventions that aim to appropriately increase antibiotic use in particular settings have resulted in large improvements of 15–25%, such as training community members to diagnose and treat childhood pneumonia cases with antibiotics in Nepal. [39] However, interventions that aim to decrease antibiotics use appear to generate more modest improvements–as happened in Malaysia where academic detailing resulted in 11% reduction in antibiotic use for upper respiratory tract infection.[40] The effect sizes on antibiotic prescribing quality of many of the individual policies examined in this study were modest. However, the overall effect size of multiple policies on the quality of antibiotic use in the treatment of acute diarrhoea and acute respiratory tract infection was large and comparable with the largest intervention effects reported elsewhere.[8, 23] A greater effect size with multiple interventions, as compared to a single intervention, has been described in several literature reviews.[8–9, 23, 41–42]

In our view the policies we have highlighted in these analyses reported here represent a blueprint for a national strategy to promote rational use of antibiotics–a critical component in the fight to contain anti-microbial resistance.

### Limitations

We relied on self-reported information on policy implementation. Causality cannot be ascribed to the associations we have highlighted and we cannot be confident that effects associated with one policy are not due to another policy co-intervention. The small sample sizes prevented multi-variable analyses. But these limitations apply to the whole field. In the absence of carefully collected longitudinal data at national level it is not possible to perform time series analysis, which represents a gold standard in policy evaluation.

We acknowledge that a multiple correction adjustment, such as Bonferroni, would render some of our findings statistically insignificant. However, our intention was not to test multiple hypotheses, particularly regarding the relative impact of the different policies. Our emphasis here is in identifying patterns in the data rather than emphasising statistical significance. The data themselves have serious limitations, which we address below. Nevertheless, they are the only comprehensive data available to address these important questions. The largest estimated policy effects were seen with the disease-specific antibiotic use measures (diarrheal disease and upper respiratory infections), and the strongest correlations with numbers of implemented policies were also seen for these outcomes. Taken together these suggest that our overall findings are quite robust.

We have previously documented other limitations of the data-sets used here.[22] Briefly, policies reported as implemented by MOHs may not have been implemented. The antibiotic use survey data for countries may not be generalizable, having come from individual published surveys although care was taken only to include surveys that followed minimum standards.[25] However, misclassification of policy implementation and inaccurate antibiotic use data would tend to weaken correlations seen between policy and antibiotic use. We were concerned that some countries that implemented multiple policies would feel under pressure to report better and lower antibiotic use. However, data on policy implementation came from MOHs and antibiotic use data from independently conducted surveys which reduces but does not eliminate the chance of biased reporting. Results were confined to the public sector due to lack of antibiotic use data in the private sector. Nevertheless, many of the policies may spill over into the private sector as doctors in low and middle income countries often have both public and private practices. We recognise our dependence on rather old data. We addressed this in our previous report.[22] In our view the findings here still have relevance as the essential medicines policy landscape has changed little in the last 10 years and the policies studied here are still recommended for widespread use.

We also need to consider the potential adverse effects of the policies designed to reduce dependence on antibiotics. It is possible that when policies target and reduce one particular antibiotic prescribing behaviour, another inappropriate behaviour is substituted. For example, increased antibiotic consumption has occurred after campaigns to reduce injection use.[43] Thus, it could be that reduction of antibiotic use for upper respiratory tract infection and acute diarrhoea could be accompanied by increased antibiotic use for other conditions or doctors simply changing the diagnoses to justify continued prescribing of an antibiotic. This may account for the lack of effect of policies on the overall use of antibiotics.

Our findings should be interpreted with a degree of caution. It may be that countries with more functional and efficient health systems are more likely to implement policies to promote better quality use of antibiotics. Better quality of antibiotic use was found in countries with higher economic status and this may be because of healthier populations, better health systems and/or more policy implementation

### Conclusions

This is the first study to evaluate the impact of essential medicines policies recommended in the WHO Global Strategy to contain AMR and the WHO Global Action Plan 2015 on antibiotic use in multiple countries. The study shows in detail how different policies and numbers of policies impact on different indicators of antibiotic usage. The findings confirm that countries implementing these policies have less inappropriate use of antibiotics. This finding is important in the fight to contain anti-microbial resistance. It is critical that much more international attention is focused on implementing essential medicine policies to reduce antibiotic use in low and middle income countries.

### Disclaimer

Kathleen Anne Holloway is a staff member of the World Health Organization. The authors alone are responsible for the views expressed in this publication and they do not necessarily represent the decisions or policies of the World Health Organization.

## Supporting Information

S1 Table Reported antibiotic policy implementation by country. (XLSX)

S2 Table Individual antibiotic use measures by country plus references. (XLS)
